# Glucagon-like peptide receptor agonists (GLP-1RAs) as cardiovascular risk modifiers in obstructive sleep apnea and obesity: a real-world study

**DOI:** 10.1007/s44470-026-00110-x

**Published:** 2026-07-01

**Authors:** Jaeun Ahn, Junmin Song, Kyle Admire, Yu Chang, Kuan-Yu Chi, Nathaniel T. Gordon, Joshua M. Sill

**Affiliations:** 1 Department of Medicine, Eastern Virginia Medical School at Old Dominion University, 825 Fairfax Avenue, Norfolk, VA 23507 USA; 2 Department of Medicine, Jacobi Medical Center, Albert Einstein College of Medicine, Bronx, NY USA; 3 Section of Neurosurgery, Department of Surgery, College of Medicine, National Cheng Kung University Hospital, National Cheng Kung University, Tainan City, Taiwan

**Keywords:** Obstructive sleep apnea, Glucagon-like peptide-1 receptor agonists, GLP-1 RA, Cardiovascular risk

## Abstract

**Introduction:**

Obstructive sleep apnea (OSA) is a well-established risk factor for cardiovascular disease. Glucagon-like peptide-1 receptor agonists (GLP-1RAs) have demonstrated potential in reducing cardiovascular risk and improving OSA severity. However, a significant knowledge gap remains regarding the impact of GLP-1RAs on cardiovascular events in OSA patients.

**Methods:**

We examined patients with a BMI over 30 diagnosed with OSA between January 2010 and November 2021 without a previous diagnosis of heart failure, pulmonary hypertension, or myocardial infarction. Two cohorts were created: those prescribed GLP-1RAs within 1 year of their OSA diagnosis and patients who were never prescribed GLP-1RAs. Propensity score matching was performed using TriNetX software with greedy nearest neighbor methodology. Cox proportional hazards analyses were conducted over a 3-year follow-up period. The primary outcome was newly diagnosed heart failure. Secondary outcomes included pulmonary hypertension, death from all causes, ischemic stroke, and acute myocardial infarction (AMI).

**Results:**

We included 18,774 patients in the GLP-1RA cohort and 847,137 patients in the non-GLP-1RA cohort. Propensity score matching resulted in 18,523 patients remaining in each cohort, which were well-matched in demographics, comorbidities, medications, BMI, and HbA1c. After 3 years, the GLP-1RA cohort had a significantly lower risk of newly developed heart failure (HR, 0.76; 95% CI, 0.71–0.82), pulmonary hypertension (HR, 0.67; 95% CI, 0.59–0.75), AMI (HR, 0.76; 95% CI, 0.67–0.86), and death from all causes (HR, 0.57; 95% CI, 0.51–0.63).

**Conclusion:**

GLP-1RAs were associated with reduced risks of heart failure, pulmonary hypertension, AMI, and death from all causes in obese OSA patients. Prospective studies are needed for validation.

**Brief summary:**

GLP-1 receptor agonists (GLP-1RAs) have shown promising potential in reducing cardiovascular risk and alleviating the severity of obstructive sleep apnea (OSA), a well-known risk factor for cardiovascular disease. However, a significant knowledge gap remains regarding the impact of GLP-1RAs on cardiovascular events specifically in patients with OSA.

With 3 years of follow-up, the GLP-1RAs reduced newly developed heart failure by 24%, pulmonary hypertension by 33%, acute myocardial infarction by 24%, and death by 43%, with notable early separation in heart failure and all-cause mortality. This finding supports the potential for broader use of GLP-1RAs in this population, not only to improve OSA severity, but also to reduce cardiovascular risk.

## Introduction

Obstructive sleep apnea (OSA) is a well-established risk factor for cardiovascular disease [1]. Glucagon-like peptide-1 receptor agonists (GLP-1RAs) have shown promising potential in reducing cardiovascular risk in patients with diabetes, and there is emerging evidence suggesting that GLP-1RAs may help alleviate OSA symptoms [2–6]. In the SURMOUNT-OSA trial, treatment with tirzepatide, a dual GLP-1 receptor and glucose-dependent insulinotropic polypeptide (GIP) agonist, led to improvements in the Apnea–Hypopnea Index (AHI), high-sensitivity C-reactive protein (hsCRP), and systolic blood pressure, indicating potential cardiovascular benefits [5]. A recent meta-analysis [7, 8] demonstrated that both GLP-1RAs and tirzepatide significantly reduce the severity of OSA, including improvements in the AHI, and emerging real-world evidence suggests that tirzepatide may confer greater cardiometabolic benefit than selective GLP-1RAs [9, 10]. However, evidence regarding the impact of selective GLP-1 receptor agonists on the risk of incident heart failure in patients with obstructive sleep apnea remains limited. Therefore, we leveraged a large-scale global healthcare database to examine the association between GLP-1RA use and subsequent development of heart failure in this high-risk population.

## Methods

We conducted a propensity score-matched retrospective cohort study using aggregate data from the TriNetX platform [11]. TriNetX Global Collaborative Network includes over 160 million patients from more than 120 healthcare organizations worldwide, encompassing both inpatient and outpatient settings [11]. It complies with the General Data Protection Regulation and the Health Insurance Portability and Accountability Act [12, 13]. We included patients diagnosed with OSA between January 2010 and November 2021 with a BMI over 30. Patients with a diagnosis of heart failure, pulmonary hypertension, or myocardial infarction prior to the OSA diagnosis were excluded. Two cohorts were created: (1) the GLP-1RA user cohort, consisting of patients prescribed GLP-1RAs (semaglutide, liraglutide, or dulaglutide) within 1 year of their OSA diagnosis, and (2) the non-GLP-1RA user cohort, comprising OSA patients who were never prescribed any type of GLP-1RA before or after the OSA diagnosis. The index date was defined as the date of OSA diagnosis for all patients, with follow-up beginning uniformly at this date for both cohorts. Tirzepatide, a dual GLP-1/GIP receptor agonist, was not included in the GLP-1RA exposure definition because it was not FDA-approved during the study period (2010–2021) and its dual mechanism of action differs from GLP-1 selective agents. Within the cohorts, baseline characteristics were matched using propensity score matching by using the greedy nearest neighbor method. The primary outcome was newly diagnosed heart failure, and secondary outcomes were pulmonary hypertension, death from all causes, ischemic stroke and acute myocardial infarction (AMI), each defined using International Classification of Diseases Version 10 (ICD-10) codes (Table 1). These ICD-10-based outcome definitions have been previously used in TriNetX-based cardiovascular studies [14, 15]. Cox proportional hazards analyses were conducted over a 3-year follow-up period. Statistical significance was defined as a two-sided *P*-value less than 0.05. For baseline characteristic matching, a standardized mean difference (SMD) of less than 0.2 was considered a small difference. All analyses were performed using the built-in TriNetX software and R version 4.4.0.

**Table 1:** ICD-10 code-based definitions of baseline condition and key outcomes

Condition	ICD-10 codes	Diagnosis
Heart failure	I50	Heart failure
Acute myocardial infarction	I21	Acute myocardial infarction
	I22	Subsequent ST elevation (STEMI) and non-ST elevation (NSTEMI) myocardial infarction
Pulmonary hypertension	I27.0	Primary pulmonary hypertension
	I27.2	Other secondary pulmonary hypertension
Ischemic stroke	I63	Cerebral infarction
	I67.82	Cerebral ischemia
Obstructive sleep apnea	G47.33	Obstructive sleep apnea

## Results

**Fig. 1:**
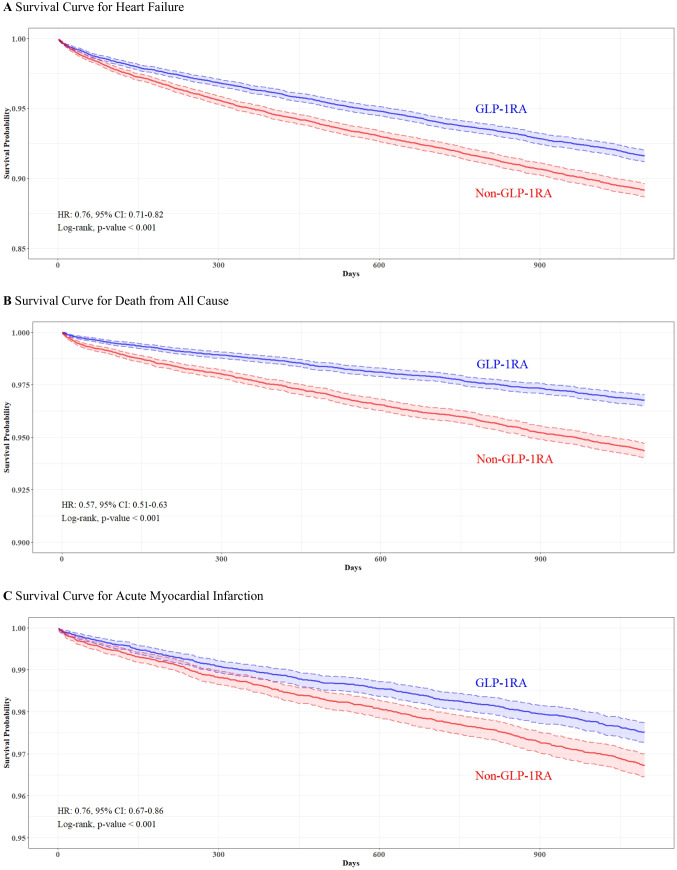

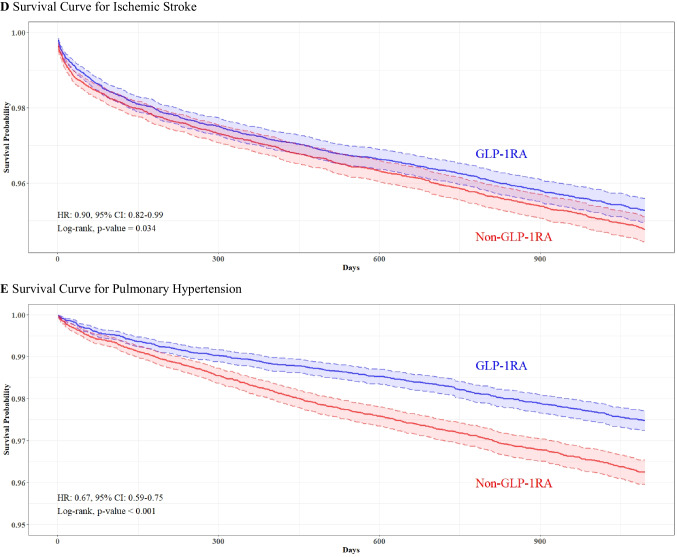
Kaplan–Meier survival curves for heart failure (**A**), death from all causes (**B**), acute myocardial infarction (**C**), ischemic stroke (**D**), and pulmonary hypertension (**E**)

We included 18,774 patients in the GLP-1RA cohort and 847,137 patients in the non-GLP-1RA cohort. Before matching, the cohorts primarily consisted of White patients (GLP-1RA users vs. non-users: 69.8% vs. 72.8%). The GLP-1RA group had a higher proportion of females (54.2% vs. 46.0%), a higher mean hemoglobin A1c level (mean HbA1c, 7.8 vs. 6.4), and a higher mean BMI (40.8 vs. 38.7). After propensity score matching, each cohort included 18,523 patients. Demographic and baseline characteristics are summarized in Table 2. The matched cohorts were well balanced in terms of demographics (mean age, 54.8 vs. 55.0 years; White, 69.8% vs. 69.4%; male, 45.9% vs. 45.5%), comorbidities, medications, body mass index (mean BMI, 40.8 vs. 40.7), and HbA1c (mean, 7.8 vs. 7.7). Among GLP-1RA users, most received liraglutide (51.7%), followed by dulaglutide (35.0%) and semaglutide (19.1%). After 3 years of follow-up, the GLP-1RA cohort had a significantly lower risk of death from all causes (550 vs. 945 cases; hazard ratio [HR], 0.57; 95% confidence interval [CI], 0.51–0.63; *P* < 0.001), newly diagnosed heart failure (1416 vs. 1791 cases; HR, 0.76; 95% CI, 0.71–0.82; *P* < 0.001), newly diagnosed pulmonary hypertension (424 vs. 617 cases; HR, 0.67; 95% CI, 0.59–0.75; *P* < 0.001), newly diagnosed ischemic stroke (810 vs. 877 cases; HR, 0.90; 95% CI, 0.82–0.99; *P* = 0.034) and newly diagnosed AMI (417 vs. 535 cases; HR, 0.76; 95% CI, 0.67–0.86; *P* < 0.001) compared to the non-user cohort (Fig. 1 and Table 3). In an exploratory subgroup analysis by medication class, each GLP-1RA was independently associated with a significantly lower risk of newly diagnosed heart failure: dulaglutide (HR, 0.75; 95% CI, 0.66–0.84; *P* < 0.001), semaglutide (HR, 0.83; 95% CI, 0.70–0.99; *P* = 0.043), and liraglutide (HR, 0.73; 95% CI, 0.67–0.81; *P* < 0.001).

**Table 2:** Patient baseline characteristics before and after propensity score matching

Characteristic Name	Before propensity score matching			After propensity score matching		
	GLP-1 RA group (*n* = 18,774)	Non-GLP-1 RA group (*n* = 847,137)	SMD	GLP-1 RA group (*n* = 18,523)	Non-GLP-1 RA group (*n* = 18,523)	SMD
Basic demographics						
Age at index, mean	54.7 ± 11.8	52.9 ± 14.9	0.13	54.8 ± 11.8	55.0 ± 13.2	0.01
Male	8592 (45.8)	457,187 (54.0)	0.16	8503 (45.9)	8425 (45.5)	0.01
White	13,102 (69.8)	617,047 (72.8)	0.07	12,929 (69.8)	12,864 (69.4)	0.01
Black or African American	3132 (16.7)	133,719 (15.8)	0.02	3087 (16.7)	3168 (17.1)	0.01
Hispanic or Latino	1420 (7.6)	53,546 (6.3)	0.05	1402 (7.6)	1359 (7.3)	0.01
Asian	232 (1.2)	10,001 (1.2)	0.01	230 (1.2)	210 (1.1)	0.01
Exam values						
Hemoglobin A1c (HbA1c), mean	7.8 ± 1.9	6.4 ± 1.4	0.87	7.8 ± 1.9	7.7 ± 1.8	0.05
Body Mass Index (BMI), mean	40.8 ± 7.7	38.7 ± 7.3	0.29	40.8 ± 7.7	40.7 ± 7.5	0.01
Diabetes medications						
Sodium-glucose co-transporter 2 inhibitors	3220 (17.2)	5201 (0.6)	0.61	2982 (16.1)	2428 (13.1)	0.08
Sulfonylureas	3821 (20.4)	23,702 (2.8)	0.57	3735 (20.2)	3842 (20.7)	0.01
Thiazolidinediones	1019 (5.4)	4854 (0.6)	0.29	982 (5.3)	978 (5.3)	< 0.01
Insulin	8854 (47.2)	74,192 (8.8)	0.95	8643 (46.7)	8696 (46.9)	0.01
Cardiovascular medications						
Angiotensin-converting enzyme inhibitors	6233 (33.2)	127,274 (15.0)	0.43	6122 (33.1)	6350 (34.3)	0.03
Angiotensin II receptor blockers	4939 (26.3)	90,068 (10.6)	0.41	4825 (26.0)	4953 (26.7)	0.02
Beta-blockers	6252 (33.3)	182,330 (21.5)	0.27	6179 (33.4)	6534 (35.3)	0.04
Loop diuretics	2454 (13.1)	58,980 (7.0)	0.2	2422 (13.1)	2573 (13.9)	0.02
HMG CoA reductase inhibitors	10,807 (57.6)	190,761 (22.5)	0.77	10,586 (57.2)	10,877 (58.7)	0.03
Underlying comorbidities						
Type 2 diabetes	15,419 (82.1)	184,127 (21.7)	1.52	15,168 (81.9)	15,622 (84.3)	0.07
Hypertension	14,161 (75.4)	459,274 (54.2)	0.46	13,950 (75.3)	14,193 (76.6)	0.03
Ischemic heart diseases	2452 (13.1)	81,888 (9.7)	0.11	2420 (13.1)	2551 (13.8)	0.02
Cerebrovascular diseases	923 (4.9)	36,944 (4.4)	0.03	918 (5.0)	909 (4.9)	< 0.01
Chronic lung diseases	4143 (22.1)	174,669 (20.6)	0.04	4092 (22.1)	4160 (22.5)	0.01
Tobacco use	547 (2.9)	22,066 (2.6)	0.02	538 (2.9)	539 (2.9)	< 0.01

*HMG CoA* β-hydroxy β-methylglutaryl-coenzyme A, *GLP-1 RA* glucagon-like peptide-1 receptor agonist, *SMD* standardized mean difference,

**Table 3:** Primary and key secondary outcomes

	GLP-1 RA cohort (total *N* = 18,523)	Non-GLP-1 RA cohort (total *N* = 18,523)	Hazard ratio (95% CI)	*P*-value (log-ranked)	*P*-value (proportionality)
Primary outcome: heart failure	1416	1791	0.761 (0.710–0.816)	< 0.001	0.161
Secondary outcome:					
All-cause mortality	550	945	0.565 (0.509–0.628)	< 0.001	0.490
Acute myocardial infarction	417	535	0.757 (0.666–0.860)	< 0.001	0.879
Ischemic stroke	810	877	0.902 (0.820–0.992)	0.034	0.626
Pulmonary hypertension	424	617	0.665 (0.588–0.753)	< 0.001	0.307

## Discussion

In this large retrospective cohort study with a 3-year follow-up, we found that the use of GLP-1RA was associated with a significantly lower risk of developing new-onset heart failure (HR: 0.76) among patients diagnosed with obstructive sleep apnea. Furthermore, secondary cardiovascular outcomes, including new diagnoses of pulmonary hypertension and myocardial infarction, were also significantly reduced in patients receiving these agents. These findings suggest that GLP-1RAs may serve as effective cardiovascular risk modifiers in this high-risk population. Notably, the Kaplan–Meier survival curve of the primary and secondary outcomes except for pulmonary hypertension revealed early separation of survival between the two groups, which suggests direct cardiovascular benefits of GLP-1RAs, beyond weight loss, on an obese OSA population.

Our results are aligned with prior clinical trial evidence supporting the cardiovascular benefits of GLP-1RA in patients with type 2 diabetes mellitus, particularly with agents such as liraglutide, semaglutide, and dulaglutide [3]. However, while their efficacy in improving glycemic control and reducing cardiovascular events in diabetic populations is well documented, their role in patients with obstructive sleep apnea and coexisting obesity has been far less explored [3]. Previous research addressing cardiovascular risk in this setting has predominantly focused on the use of positive airway pressure (PAP) therapy. For example, a study by O’Donnell et al. reported cardiovascular benefits associated with PAP use, though the study was limited by a small sample size of only 30 participants [16]. In contrast, our study includes a much larger cohort and suggests that GLP-1RA may represent an alternative or adjunctive treatment option, particularly in patients with obesity who often struggle with adherence to PAP therapy.

The potential for GLP-1RAs to address both metabolic and cardiovascular risk factors offers a complementary therapeutic strategy alongside PAP therapy in patients with OSA. Prior randomized trials, such as SUSTAIN-6 [2], demonstrated a nonsignificant trend toward reduced nonfatal myocardial infarction with semaglutide. In this context, our findings from a large real-world cohort suggest an association between GLP-1RA use and lower rates of cardiovascular outcomes; however, these results should be interpreted cautiously given the observational nature of the analysis. Potential mechanisms underlying these associations may include improvements in endothelial function, reductions in systemic inflammation, and hemodynamic effects related to weight loss [2, 4, 6]. Supporting this biologic plausibility, the recent SURMOUNT-OSA trial evaluating tirzepatide, a dual GLP-1 and GIP receptor agonist, demonstrated improvements in both AHI and hsCRP levels, suggesting that incretin-based therapies may exert multifaceted cardiometabolic effects in patients with OSA [5].

Our study adds to this growing body of evidence by indicating that GLP-1RA alone may confer cardiovascular protection in patients with obstructive sleep apnea and obesity. Importantly, the strengths of our study include the use of a large and diverse real-world dataset, the application of propensity score matching to minimize confounding, and a long-term follow-up period, which enhances both statistical power and generalizability. However, several limitations should be acknowledged. Our database lacked granular clinical data such as severity of OSA (e.g., AHI), diabetes duration, adherence to PAP therapy, PAP claims data, and longitudinal changes in body weight. These unmeasured variables are clinically important. More severe OSA may confer higher baseline cardiovascular risk and prompt more aggressive PAP use, which itself carries independent cardiovascular benefit. Diabetes duration may further stratify residual cardiovascular risk beyond what HbA1c matching captures. Differential weight trajectories between cohorts, expected given GLP-1RA’s weight-lowering effects, may have also partly contributed to the observed outcome differences. Additionally, unmeasured lifestyle factors such as alcohol consumption and exercise frequency were not captured in the TriNetX platform and may represent sources of residual confounding. Furthermore, reliance on administrative diagnosis codes introduces the potential for misclassification bias, though such errors are likely nondifferential and would bias results toward the null. Lastly, the retrospective observational nature of our study precludes causal inference, and prospective validation is warranted.

In conclusion, our findings raise important clinical questions about the role of GLP-1RA in managing cardiovascular risk beyond traditional metabolic indications. Future prospective studies should incorporate detailed clinical data, including serial assessments of sleep apnea severity, adherence to airway pressure therapy, and body weight trajectories. Randomized-controlled trials specifically evaluating the cardiovascular impact of GLP-1RA in patients with obstructive sleep apnea, particularly those with obesity, are needed to confirm these findings and to further elucidate underlying mechanisms.

## Data Availability

Data used in this study were obtained from the TriNetX research network. Restrictions apply to the availability of these data, which were used under license for the current study and are therefore not publicly available. Data supporting this study may be shared upon reasonable request to the corresponding author.

## Abbreviations

AHI: Apnea-hypopnea index
AMI: Acute myocardial infarction
BMI: Body mass index
CI: Confidence interval
GIP: Glucose-dependent insulinotropic polypeptide
GLP-1RA: Glucagon-like peptide-1 receptor agonist
HR: Hazard ratio
hsCRP: High-sensitivity C-reactive protein
ICD-10: International classification of diseases, 10th revision
OSA: Obstructive sleep apnea
PAP: Positive airway pressure
SMD: Standardized mean difference

## References

[1] Veasey SC, Rosen IM. Obstructive sleep apnea in adults. N Engl J Med. 2019;380(15):1442–9. 10.1056/NEJMcp1816152.30970189 10.1056/NEJMcp1816152

[2] Marso SP, Bain SC, Consoli A, et al. Semaglutide and cardiovascular outcomes in patients with type 2 diabetes. N Engl J Med. 2016;375(19):1834–44. 10.1056/NEJMoa1607141.27633186 10.1056/NEJMoa1607141

[3] Kalyani RR. Glucose-lowering drugs to reduce cardiovascular risk in type 2 diabetes. N Engl J Med. 2021;384(13):1248–60. 10.1056/NEJMcp2000280.33789013 10.1056/NEJMcp2000280

[4] Gerstein HC, Colhoun HM, Dagenais GR, et al. Dulaglutide and cardiovascular outcomes in type 2 diabetes (REWIND): a double-blind, randomised placebo-controlled trial. Lancet. 2019;394(10193):121–30. 10.1016/S0140-6736(19)31149-3.31189511 10.1016/S0140-6736(19)31149-3

[5] Gu C, Bernstein N, Mittal N, et al. Potential therapeutic targets in obesity, sleep apnea, diabetes, and fatty liver disease. J Clin Med. 2024. 10.3390/jcm13082231.38673503 10.3390/jcm13082231PMC11050527

[6] Marso SP, Daniels GH, Brown-Frandsen K, et al. Liraglutide and cardiovascular outcomes in type 2 diabetes. N Engl J Med. 2016;375(4):311–22. 10.1056/NEJMoa1603827.27295427 10.1056/NEJMoa1603827PMC4985288

[7] Drager LF. A meta-analysis of glucagon-like peptide 1 receptor and dual agonists for the treatment of obstructive sleep apnea: end of the story or just the beginning? Sleep. 2025. 10.1093/sleep/zsaf004.39758029 10.1093/sleep/zsaf004

[8] Li M, Lin H, Yang Q, et al. Glucagon-like peptide-1 receptor agonists for the treatment of obstructive sleep apnea: a meta-analysis. Sleep. 2025. 10.1093/sleep/zsae280.39626095 10.1093/sleep/zsae280

[9] Wu JY, Chen CC, Ling Tu W, et al. Clinical impact of tirzepatide on patients with OSA and obesity. Chest. 2025;168(3):785–96. 10.1016/j.chest.2025.03.030.40254150 10.1016/j.chest.2025.03.030

[10] Henney AE, Riley DR, Anson M, et al. Comparative efficacy of tirzepatide, liraglutide, and semaglutide in reduction of risk of major adverse cardiovascular events in patients with obstructive sleep apnea and type 2 diabetes: real-world evidence. Ann Am Thorac Soc. 2025;22(7):1042–52. 10.1513/AnnalsATS.202409-923OC.40590655 10.1513/AnnalsATS.202409-923OC

[11] Palchuk MB, London JW, Perez-Rey D, et al. A global federated real-world data and analytics platform for research. JAMIA Open. 2023;6(2):ooad035. 10.1093/jamiaopen/ooad035.37193038 10.1093/jamiaopen/ooad035PMC10182857

[12] Chiang CH, Song J, Chang YC, et al. Glucagon-like peptide 1 receptor agonists and venous thromboembolism in type 2 diabetes: a target trial emulation. Blood Adv. 2025. 10.1182/bloodadvances.2025015871.39908569 10.1182/bloodadvances.2025015871PMC12142527

[13] Chiang CH, Song J, Chi KY, et al. Glucagon-like peptide-1 agonists reduce cardiovascular events in cancer patients on immune checkpoint inhibitors. Eur J Cancer. 2025;216:115170. 10.1016/j.ejca.2024.115170.39709670 10.1016/j.ejca.2024.115170

[14] Chi KY, Song J, Desphande S, et al. GLP-1 RA use and major adverse cardiovascular events in patients with monoclonal gammopathy of undetermined significance. JAMA Netw Open. 2025;8(6):e2517541. 10.1001/jamanetworkopen.2025.17541.40587132 10.1001/jamanetworkopen.2025.17541PMC12210086

[15] Song J, Cali Daylan AE, Chi KY, et al. Association between GLP-1 receptor agonists and incidence of lung cancer in treatment-naïve type 2 diabetes. J Gen Intern Med. 2025;40(4):973–6. 10.1007/s11606-024-09076-z.39365528 10.1007/s11606-024-09076-zPMC11914447

[16] O’Donnell C, Crilly S, O’Mahony A, et al. Continuous positive airway pressure but not GLP1-mediated weight loss improves early cardiovascular disease in obstructive sleep apnea: a randomized proof-of-concept study. Ann Am Thorac Soc. 2024;21(3):464–73. 10.1513/AnnalsATS.202309-821OC.38096106 10.1513/AnnalsATS.202309-821OC

